# Changing the Tendency to Integrate the Senses

**DOI:** 10.3390/brainsci12101384

**Published:** 2022-10-13

**Authors:** Saul I. Quintero, Ladan Shams, Kimia Kamal

**Affiliations:** 1 Department of Psychology, University of California, Los Angeles, CA 90095, USA; saulivan@g.ucla.edu (S.I.Q.); kkamal@g.ucla.edu (K.K.); 2 Department of Bioengineering, University of California, Los Angeles, CA 90089, USA; 3 Neuroscience Interdepartmental Program, University of California, Los Angeles, CA 90089, USA

**Keywords:** binding tendency, probability of common cause, integration tendency, multisensory integration, coupling prior, multisensory learning, multisensory plasticity, multisensory binding, crossmodal binding, sensory binding, integration learning

## Abstract

Integration of sensory signals that emanate from the same source, such as the visual of lip articulations and the sound of the voice of a speaking individual, can improve perception of the source signal (e.g., speech). Because momentary sensory inputs are typically corrupted with internal and external noise, there is almost always a discrepancy between the inputs, facing the perceptual system with the problem of determining whether the two signals were caused by the same source or different sources. Thus, whether or not multisensory stimuli are integrated and the degree to which they are bound is influenced by factors such as the prior expectation of a common source. We refer to this factor as the tendency to bind stimuli, or for short, binding tendency. In theory, the tendency to bind sensory stimuli can be learned by experience through the acquisition of the probabilities of the co-occurrence of the stimuli. It can also be influenced by cognitive knowledge of the environment. The binding tendency varies across individuals and can also vary within an individual over time. Here, we review the studies that have investigated the plasticity of binding tendency. We discuss the protocols that have been reported to produce changes in binding tendency, the candidate learning mechanisms involved in this process, the possible neural correlates of binding tendency, and outstanding questions pertaining to binding tendency and its plasticity. We conclude by proposing directions for future research and argue that understanding mechanisms and recipes for increasing binding tendency can have important clinical and translational applications for populations or individuals with a deficiency in multisensory integration.

## 1. Introduction

Although we are generally not aware of the multitude of sensory signals that our nervous systems continuously process, our experience of the world is strikingly multisensory, providing a rich array of sensory signals at any given moment. The research in the last two decades has shed light on the variety of advantages that multisensory processing provides in human perception compared to unisensory processing. It is now well established that multisensory information enhances the accuracy of detection, identification, discrimination [1], improves reliability (i.e., precision) of the sensory estimates [2], improves processing speed [3], and provides more complete information about the objects around us [4].

Multisensory processing involves two steps: causal inference followed by source estimation. As an example, consider having a Zoom meeting with a few people in a gallery view mode. Imagine that two or more people speak at the same time. Your perceptual system must first determine which voices and faces belong to the same person, and then for those voices and faces that are inferred to belong to the same person, it needs to integrate the lip movements and vocal information to determine the best estimate of what the person is saying. For those faces that are inferred not to be talking or for those voices that may not be visible (not on the screen or not in the fovea), it needs to keep them separate for the best estimate of what the individual is doing/saying. The process of determining which faces and voices belong to the same person/source and which ones do not is referred to as causal inference. This process is followed by an estimation of the variable of interest, speech in this case, as informed by the inferred causal structure.

Integration of sensory inputs in neuro-typically developed adults seems to be statistically optimal with sensory inputs getting combined, each weighted by their respective reliability [5,6,7]. Developmental studies of multisensory integration suggest that generally by adolescence multisensory integration reaches its adult stage, which is statistically optimal [8,9]. The reliability of a sensory signal depends on the quality of the input. For example, the visual input we receive about cars driving on the street on a sunny day is generally high fidelity, but the same scene on a foggy day could produce a noisy and low-fidelity visual input. This aspect of signal reliability depends on external noise and is completely out of our control. The other aspect determining the reliability of a sensory signal is the precision of the sensory representation of the input. This depends on the internal noise, i.e., the stochasticity in the neural representations, and may be improved by extensive training [10]. However, in general, this type of precision is fairly stable. Traditionally, studies of perceptual learning have focused on this type of learning: the training that aims to improve the precision of unisensory perceptual encoding of features.

Here we focus on learning related to the first stage of processing in multisensory perception: causal inference. Although multisensory causal inference occurs at every moment of our awake lives, and it appears to be quite plastic (as we discuss below), surprisingly few studies have systematically explored this topic, and several questions remain open regarding this very important computational process in the brain.

Returning to the example above, the process of inferring whether a given voice and a given face video have a common cause, which we refer to as causal inference, depends on two factors: (a) how congruent the two sensory inputs are (in time, space, structure or semantics, for example, the phonology of vocal and lip-reading stimuli), and (b) in general, how often a face video and voice audio are generated by the same source. Given the same stimuli (visual and auditory) two different observers may perceive the causal structure differently depending on their bias or prior expectation of a common source, i.e., depending on the factor (b). In the Bayesian framework, factor (a) corresponds to the likelihood functions of the sensory stimuli, and factor (b) corresponds to the prior probabilities of events in the world. Prior expectation of a common cause is shaped by prior experience (how often in our lifetime we have experienced similar videos and audios to have a common cause), and/or explicit knowledge about an event/setting (e.g., if we are told by a moderator that person X is now speaking; or in the context of an experiment a bias can be produced explicitly or implicitly by the experimenter). Some priors may also be at least partially hard-wired by evolution. However, generally speaking, the priors reflect the statistics and regularities of the events in the environment that are learned by the nervous system as a result of the efficient statistical learning mechanisms of the perceptual system.

In the absence of systematic and explicit training, the prior bias for binding the senses appears to be fairly stable, at least within the course of about 7 days [11]. The larger the bias for binding (or prior probability of a common cause), the more frequently the perceptual system integrates the inputs across the senses and the more tolerant the perceptual system would be to discrepancies between the sensory inputs, i.e., factor (a) would play a smaller role in the causal inference process.

Can the prior tendency to bind be modified by learning? And if so, how? The necessary and sufficient conditions for this type of learning are not well understood and the neural mechanisms are not well studied. However, in recent years, there have been several studies that have probed these questions. Here we review these studies, highlight the important open questions, and suggest directions for future research. Multisensory training/experience/encoding can also result in improved unisensory processing. We have discussed these phenomena elsewhere [1,12] and will not discuss this topic here.

## 2. Multisensory Binding Tendency

**Figure 1:**
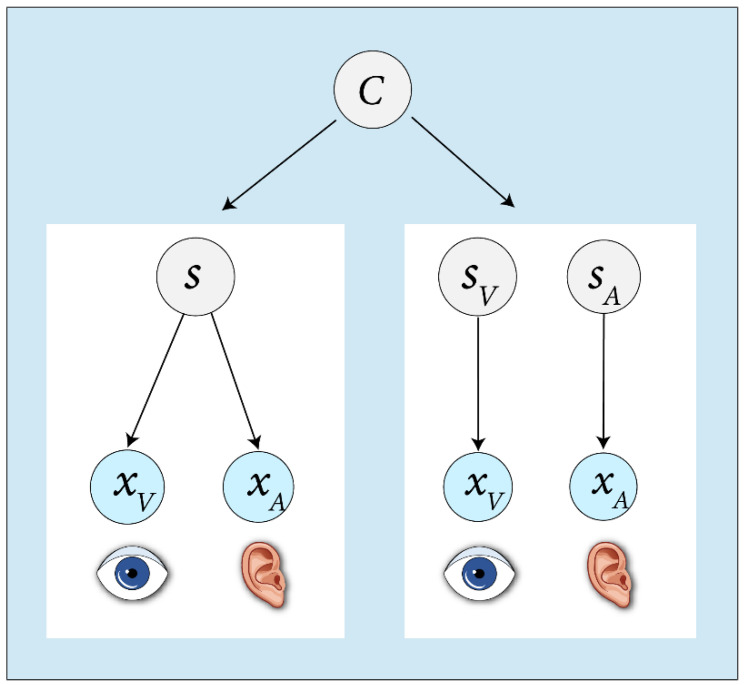
The generative model of Bayesian causal inference [5]. Two causal structures can give rise to auditory and visual stimuli $x_{A}$ and $x_{V}$ . Either a common cause ( s) has produced the stimuli (C=1) , or independent causes $s_{A}\;$ and $\;s_{V}$ , have produced the two stimuli (C=2) .

Because both the relative reliability of unisensory representations and the prior probability of common cause, or binding tendency as we call it henceforth, influence the inference of causal structure [11], it is important to use rigorous methods that allow characterization or quantification of each of these factors without being confounded by the other. The Bayesian causal inference (BCI) model of multisensory perception [13,14,15] (Figure 1) provides a rigorous quantification of both unisensory reliabilities and the tendency to bind the senses for each individual observer. Indeed, BCI has been used by a few recent learning studies in order to disentangle the plasticity in binding tendency and the plasticity in unisensory parameters. In the following sections, we first review these studies. We will then discuss studies that have not explicitly or conclusively disentangled the binding tendency and unisensory factors, but nonetheless have attempted to explore plasticity in multisensory processing. However, first, we briefly review the quantification of binding tendency in the framework of BCI.

BCI is a normative Bayesian model that makes an inference about the causal structure of sensory inputs based on the congruency between the sensory inputs and prior expectation of a common cause:

(1) $$p\left(C|x_{V},x_{A}\right)=\frac{p\left(x_{V},x_{A}|C\right)p\left(C\right)}{p\left(x_{V},x_{A}\right)}\;$$

where $p\left(C=1|x_{V},x_{A}\right)\;$ represents the posterior probability of a single cause being the source of both the current visual and auditory stimuli and $p\left(C=2|x_{V},x_{A}\right)$ represents the probability of two independent causes being the sources of the visual and auditory stimuli. $x_{V}$ and $x_{A}$ denote the visual and auditory representations, respectively.

C  is a binary variable, and therefore, the probabilities of a common cause (C=1)  and independent causes (C=2) given the current bisensory stimuli sum to 1. The closer the probability of common cause is to zero, the more likely that audiovisual signals will be segregated. Conversely, the closer it is to one, the more likely the signals will be integrated. The estimate of the variable of interest s (e.g., spatial location, temporal numerosity, syllable, etc.) is based on the inferred causal structure  C . Probability distributions $p\left(x_{A}|s_{A}\right)\;$ and $p\left(x_{V}|s_{V}\right)\;$ represent likelihood functions and are typically approximated by gaussian distributions with the mean representing the most likely representation of  s . Because the representations are corrupted by noise, the standard deviation of each likelihood distribution represents the noisiness of the visual and auditory modalities. The larger the standard deviation, the less precise each modality is in representing s . The relative precision of the two modalities influences the integration process by biasing representations to the more precise modality.  p(s)  represents the prior expectation of the source and is also approximated by a gaussian distribution with the mean $\mu_{P}\;$ and standard deviation $\sigma_{P}$ . The terms ${\hat{s}}_{\left(C=1\right)}\;$ and ${\hat{s}}_{\left(C=2\right)\;}$ represent the optimal estimate of the source under the assumption of a common cause, and independent causes, respectively.

If the sensory signals have independent causes, the optimal estimate of  s in each modality would be a weighted average of the unisensory evidence and the prior for s .

(2) $${\hat{s}}_{\left(V,C=2\right)}=\frac{\frac{x_{V}}{\sigma_{V}^{2}}+\frac{\mu_{P}}{\sigma_{P}^{2}}}{\frac{1}{\sigma_{V}^{2}}+\frac{1}{\sigma_{P}^{2}}}\;$$

(3) $${\hat{s}}_{\left(A,\;\;C=2\right)}=\frac{\frac{x_{A}}{\sigma_{A}^{2}}+\frac{\mu_{P}}{\sigma_{P}^{2}}}{\frac{1}{\sigma_{A}^{2}}+\frac{1}{\sigma_{P}^{2}}}\;$$

If the sensory signals arose from a common cause, the optimal solution would be a weighted sum of both the auditory and visual cues and the prior expectation with each weight reflecting the relative precision of the three.

(4) $${\hat{s}}_{\left(V,C=1\right)}={\hat{s}}_{\left(A,C=1\right)}=\frac{\frac{x_{V}}{\sigma_{V}^{2}}+\frac{x_{A}}{\sigma_{A}^{2}}+\frac{\mu_{P}}{\sigma_{P}^{2}}}{\frac{1}{\sigma_{V}^{2}}+\frac{1}{\sigma_{A}^{2}}+\frac{1}{\sigma_{P}^{2}}}\;$$

Of course, under normal circumstances, the nervous system has no access to the ground truth about the causal structure of the sensory inputs and must infer the causal structure from the observed sensory inputs. Therefore, the optimal estimate of environmental variables $\;s_{A}$ and $s_{V}$ , take both causal structures into account, each weighted by their respective probability (see [15] for other strategies).

(5) $${\hat{s}}_{A}=p\left(C=1|x_{A},x_{V}\right){\hat{s}}_{\left(A,C=1\right)}+p\left(C=2|x_{A},x_{V}\right){\hat{s}}_{\left(A,C=2\right)}\;$$

(6) $${\hat{s}}_{V}=p\left(C=1|x_{A},x_{V}\right){\hat{s}}_{\left(V,C=1\right)}+p\left(C=2|x_{A},x_{V}\right){\hat{s}}_{\left(V,C=2\right)}\;$$

More details about the BCI model can be found in other previous publications [5,15,16].

BCI has been shown to account for multisensory processing remarkably well in a diverse number of tasks, sensory modality combinations, and populations and has been verified at all three of Marr’s levels of analysis [17] (for a review see [18]). Therefore, BCI provides the ideal framework for a rigorous and reliable quantification of tendency to bind in a variety of tasks and domains avoiding the confounding by unisensory precision factors. The studies discussed in the next section have investigated the effect of specific multisensory training protocols on the tendency to bind multisensory stimuli and have used BCI to tease apart the effect of training on prior probability p(C=1) (also known as *Pcommon*) vs. sensory precisions $\sigma_{V}\;$ and $\sigma_{A}$ .

## 3. Multisensory Associative Learning

The tendency to bind and integrate stimuli at least in theory is influenced by a variety of factors. These include higher-level factors such as cognitive or conscious knowledge of the causal structure. For example, if the observer is made to believe (either by the instructions from the experimenter, or by the contextual information that the stimuli were caused by one object, the tendency to bind will be increased. Tendency to bind stimuli can also reflect the association between the stimuli learned by experience (for example, through statistical learning) or hard-wired by evolution.

Research has confirmed that both top-down and bottom-up mechanisms can influence the tendency to integrate cross-modal stimuli. For example, in a study the observers were made to believe that visual and haptic signals arose from the same object using a mirror, the participants integrated the stimuli despite a large spatial discrepancy between the two which would not give rise to integration otherwise [19].

Implicit learning of the associations between stimuli through passive exposure can also result in an updating of the extent to which stimuli are integrated. Research on statistical learning has established that the nervous system is equipped with a very efficient mechanism for learning statistical regularities (such as joint probabilities and conditional probabilities) in the environment based on brief passive exposure to the stimuli [20,21]. In a study investigating multisensory statistical learning, observers learned statistical regularities within the visual stream, within the auditory stream, and across the two streams all in parallel; however, the audio-visual statistical regularities were learned more efficiently than the unisensory regularities [22]. The visual and auditory stimuli were all novel and there were no pre-existing correspondences between the sounds and shapes and yet, strikingly, the associations between the shapes and sounds were learned within only a few minutes of passive exposure.

In another study, observers were exposed to objects in which the haptic stiffness and visual luminance co-varied unbeknownst to the participants [23]. These stimulus properties that are not naturally associated in nature did not get integrated prior to this exposure phase. However, subsequent to the exposure to the correlated visual-haptic properties, they were increasingly integrated suggesting that the association between the two sensory properties was learned and led to an increase in the tendency to bind/integrate. Altogether these findings indicate that the tendency to integrate sensory signals is plastic as new associations between different sensory modalities can be efficiently acquired through statistical learning, and knowledge about causal structure can rapidly modify the tendency to integrate.

## 4. Plasticity in Binding Tendency

The studies discussed above show that new associations between crossmodal features can be formed by learning. How about features that are already linked? Can the strength of association (or binding) between features that are already associated be modified by learning? Beyond mere associations, can the prior probability of a common cause for these associated features be modified through perceptual learning?

Research has shown a significant inter-individual variability in the binding tendency (i.e., the prior probability of common cause). This variability has been reported in multiple audiovisual tasks including spatial localization [11], size-weight perception [24], temporal numerosity [11,25], and audiovisual speech perception [26]. Within an individual, however, the binding tendency seems to be relatively stable at least to the span of seven days, which is the only interval investigated systematically so far [11]. In addition to being relatively stable, the tendency to bind appears to be, to a certain extent, domain specific. When the tendency to bind for various individuals during an audiovisual spatial localization task was compared to the tendency to bind during an audiovisual temporal numerosity task, there was no significant correlation between the two [25]. This suggests that integration is not governed by a single global parameter and may depend on task or stimuli demands.

Findings of a few recent studies suggest that binding tendency, despite being relatively stable and domain specific, can be *modified* by generalized perceptual learning protocols fairly quickly. The predominant assumption in the field has been that repeated exposure to congruent or incongruent stimuli would result in an increase or decrease in binding tendency, respectively, through statistical learning mechanisms (discussed in more detail in the next section).

For example, McGovern et al. [27] trained participants in a 2 Interval Forced Choice Task (2-IFC) simultaneity discrimination task. Each participant was trained in three sessions, each session consisting of 400 trials, and received feedback on their accuracy during each trial. Before and after training, participants completed a spatial localization task. The data in the pre- and post-test sessions were qualitatively compared with simulations of the BCI model with changes in different parameters. The comparisons suggested that the tendency to bind auditory and visual inputs (in the spatial task) was reduced on average after training. While the results suggest a relatively fast degree of plasticity in binding tendency, it is not clear what aspect of training gave rise to this change. The repeated exposure to the asynchronous stimuli (at times with large asynchronies) could have led to an update of the binding tendency (i.e., *Pcommon)* to better reflect the statistical distribution of auditory-visual events in the world. The audiovisual simultaneity training may also have led observers to become more sensitive to temporal discrepancies between the audiovisual stimuli which may have offered increased evidence of incongruence, ultimately reducing binding tendency via statistical learning. Although, as we discuss in the next section, it is not entirely clear if this is the main reason for the results observed by McGovern and colleagues.

The tendency to bind auditory-visual stimuli has also been shown to be altered rapidly by passive exposure, as opposed to explicit simultaneity judgment training, to auditory-visual stimulus pairings [28]. In a study by Odegaard et. al, the tendency to bind auditory and visual stimuli in a spatial localization task was quantified using BCI modeling of behavioral data before and after passive exposure to a wide range of audio-visual pairings across multiple experiments. The parameter *Pcommon* (i.e., p(C=1) as discussed earlier) quantified the tendency to bind noise bursts and flashes for each individual participant before and after an exposure phase which involved passive exposure to noise bursts and flashes with specific temporal and spatial relationships. Surprisingly, exposure to spatio-temporally congruent noise bursts and flashes did not increase the binding tendency. However, exposure to temporally congruent but spatially discrepant stimuli did and the effect size was large. After exposure to repeated spatially discrepant AV stimuli, the participants showed higher tolerance to spatial discrepancy and integrated AV stimuli more. As we will discuss in the next section, one possible explanation for these results is that, provided the frequent re-exposure of spatially discrepant but *concurrent* stimuli, the brain could be relaxing its criteria for integrating spatially discrepant audiovisual stimuli (in a predictive coding framework).

The findings of a recent study by Park and Kayser [29] are consistent with those of Odegaard et al. [28] despite the substantial differences in experimental paradigm. In this study, observers performed spatial localization of auditory and visual stimuli with variable degrees of spatial discrepancy. In one condition, the spatial discrepancies varied within a small range and up to a small degree (±26°), whereas in another condition the range was larger and capped at a larger degree (±46°). The results suggested a larger binding tendency (*Pcommon*) in the condition wherein observers were exposed to larger spatial AV discrepancies (“wide” condition). While it is in theory possible that this difference is due to a decrease in binding tendency in the small discrepancy (“narrow”) condition, it is more likely that the binding tendency increased more strongly in the large discrepancy condition compared to the small-discrepancy condition.

The findings reported by Odegaard et al. [27] and Park and Kayser [29] provide evidence that binding tendency can be increased. It remains unclear, however, why exposure to congruent stimuli did not increase the binding tendency and what type of statistical regularity would give rise to a decrease in binding tendency. It is noteworthy that even in the “incongruent” conditions both visual and auditory stimuli were presented on each trial with maximum asynchrony of 250 ms. Because the temporal binding window on average can span more than 250 ms of asynchrony between audiovisual stimuli, it is likely that more asynchrony is needed to drive binding tendency to decrease. It is possible that a complete dissociation, where audio and visual stimuli are perceived as not incongruent signals but as entirely different unisensory events, is necessary to decrease the binding tendency.

Indeed, this type of complete dissociation was tested in a recent study by Tong et al. that also provided evidence for a relatively fast malleability of auditory-visual binding tendency by passive exposure [30]. Investigating the effect of statistical learning on the tendency to integrate, participants’ ventriloquist effect was measured after passive exposure to two pairs of stimuli, one in which the auditory and visual stimuli were consistently congruent both in space and time (A1V1) and one in which the two were consistently incongruent both in space and time (A2V2). In the incongruent learning blocks, the sounds and flashes were presented either individually (‘unisensory trials’) or both were presented at different locations and with large time discrepancy. The possible temporal asynchronies in Tong et al. are significantly larger (750 ms to 1500 ms) than those used by Odegaard et al. [28]. This could render some of the ‘incongruent’ A2V2 audiovisual trials into perceived distinct *unisensory* trials. Observers’ unisensory visual and auditory localization was tested before and after the associative learning phase, and showed no change, ruling out the influence of unisensory precision changes in any post-learning changes in ventriloquism effect. The results showed a higher degree of integration for A1V1 and A1V2 (which was a stimulus pair that was not trained) compared to A2V2 and A2V1 (which was also not trained). In another experiment where the auditory stimuli during the localization phase were presented with two competing visual stimuli on opposite sides, the integration was stronger for A1 which was pulled more strongly towards V1 than A2 (or a novel sound A3). These results suggest that passive exposure to statistical regularities in auditory-visual stimuli can influence the tendency to bind at the stimulus-pair level. However, the exact nature of the modification remains unclear for multiple reasons. First, because of the absence of a baseline measurement, it is not clear whether there was an increase in binding of paired stimuli or a decrease in the binding of dissociated stimuli, or both. Moreover, because A2 and A3 were harmonics of A1, it is not clear if any generalization of the pairing that was found is related to the similarity in the representation of the sounds in the nervous system.

## 5. Learning Mechanisms Operating on Binding Tendency

As discussed in the previous section, the conditions required to elicit an update in the prior probability to bind cross-modal stimuli have not been extensively investigated by previous research. Although the ‘recipe’ for how to systematically manipulate binding tendency has not been uncovered, previous literature has endorsed the framework that repeated passive exposure to congruent audiovisual stimuli should encourage binding tendency to increase. Given the evidence that passive exposure can result in the learning of statistical relationships of audiovisual stimuli such as joint probabilities or conditional probabilities, it certainly could be the case that exposure to frequent congruent or incongruent audiovisual stimuli results in binding tendency updates to reflect the regularity of congruence or incongruence in recent perceptual history [22].

**Figure 2:**
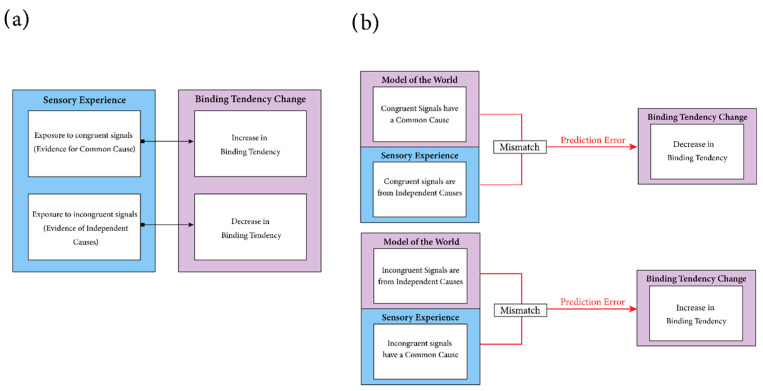
Learning mechanisms involved in binding tendency plasticity. Two possible learning mechanisms underlying the plasticity of binding tendency are illustrated. (**a**) The statistical learning mechanism: In this mechanism, every sensory event is informative and influential towards the tendency to bind. If an observer is frequently exposed to congruent audiovisual stimuli, each exposure increases the prior probability of a common cause by offering evidence of increased frequency of common-cause audiovisual stimuli. If an observer is frequently exposed to incongruent audiovisual stimuli, each exposure decreases the prior probability of a common cause. (**b**) The predictive coding mechanism. In this mechanism, only sensory events that contradict the internal generative model of the sensory environment will elicit updates in the prior probability of a common cause. For instance, if the internal generative model of the world expects that incongruent stimuli do not have a common cause, and an observer repeatedly experiences spatially incongruent but temporally congruent stimuli, these repeated sensory experiences contradict the internal model of the world and produce a model prediction-error signal. This results in a relaxation of binding tendency and an increased probability of common cause to account for the congruent stimuli that are nonetheless spatially discrepant. Adapted with permission from [28].

This statistical learning framework for binding tendency updates (Figure 2a) is supported by some of the reviewed work such as McGovern and colleagues which found that perceptual training through improving audiovisual discrimination in a simultaneity judgement task resulted in a reduction of the magnitude of multisensory illusions and was consistent with a concomitant reduction in *Pcommon*, the BCI parameter for binding tendency [27]. According to this framework, as the perceptual system tracks regularities in the environment, binding tendency shifts towards these regularities. As these shifts accumulate, binding tendency is incrementally altered. Every sensory event is informative in the sense that congruent audiovisual stimuli should offer evidence of common-cause incidence and incongruent stimuli should offer evidence of independent-causes incidence. Therefore, in McGovern and colleagues’ study [27], by improving the ability of observers to track audiovisual statistical relationships through refined temporal coding, binding tendency may decrease due to an increased sensitivity to audiovisual discrepancies which leads to the accumulation of evidence of incongruent sensory events more readily.

One alternative explanation for their results could be that there are top-down influences such as task demands or instructional biases impacting participant perceptual experience, as was discussed earlier can influence binding dynamics. For instance, participants were explicitly instructed to ignore visual stimuli rendering the auditory stimulus a more salient driving feature in the integration process. Participants could also have shifted their expectation of a common cause from implicit task demands or from subtle instructional cues that might have implied the degree to which congruent or incongruent stimuli would be presented.

In Odegaard et al.’s study [28], the effect of passive exposure to different types of auditory-visual relationships on binding tendency led to findings that could not be explained by statistical learning in that exposure to congruent and incongruent stimuli did not lead to respective increases or decreases in binding tendency. Instead, a predictive coding framework [31,32,33,34,35,36] was argued to account for their multisensory learning effects. In this framework, learning is driven by prediction errors and, therefore, events that contradict the current internal model of the world would produce the largest degree of learning. For instance, it is reasonable to expect that the nervous system’s model of the auditory-visual events holds that incongruent audiovisual stimuli tend to emerge from independent sources, and therefore, do not co-occur frequently. When repeatedly exposed to incongruent stimuli that nonetheless occur simultaneously, this generates a prediction error from the expectation that incongruent stimuli do not repeatedly co-occur.

According to the predictive coding framework (Figure 2b), this prediction error elicits an updating of the internal model of the world and along with it the prior probability of common cause. An audiovisual event that most severely violates the expectations of the internal model should elicit the strongest shifts in binding tendency. Work examining the role of early postnatal perceptual experience on integration found that rearing cats in perceptual environments in which visual and auditory stimuli were temporally coupled but spatially incongruent resulted in very similar integration to cats exposed to temporally and spatially congruent audiovisual stimuli [37]. This may suggest that temporal coincidence can have strong effects on spatial integration, more-so than the reverse. We further discuss the unique affordances of temporal or spatial congruence and incongruence in section eight. If an internal model expects temporally incongruent events to also be spatially incongruent and temporally coincident events to also be spatially congruent, then a temporally congruent but spatially incongruent event will produce the strongest prediction error. It is also important to note that it is the repeated and variable spatial discrepancies that induce a shift in binding tendency. If the audiovisual signals are discrepant in a fixed manner, a simple spatial recalibration would occur to adjust for the shift in sensory topographical representations. If the audiovisual signals are discrepant in a variable manner, a simple recalibration cannot adjust for or account for the dynamic changes in the environment. Relaxation of the model’s tolerance to spatial discrepancies could minimize the prediction error and thereby lead to a greater binding tendency.

As the greatest shift in binding tendency was found in response to exposure to temporally congruent but spatially incongruent audiovisual stimuli, Odegaard et al.’s findings support the predictive coding hypothesis. Similarly, the finding of a larger binding tendency in a condition with repeated exposure to larger AV discrepancy in Park and Kayser [29] study is inconsistent with predictions of statistical learning and more in line with the predictive coding framework.

How can we reconcile the differences in results between McGovern and colleagues [27] on the one hand and Odegaard et al. [28] and Park and Kayser, on the other hand? One possibility is that the different findings can be explained by differences in their training or testing paradigms. It is important to note that Odegaard et al. [28] employed multisensory training involving passive exposure to congruent or incongruent stimuli, and Park and Kayser employed a localization task without feedback; whereas McGovern and colleagues [27] employed an active simultaneity judgment task with trial-by-trial feedback. Moreover, the localization tasks employed to probe binding tendency before and after exposure are notably different across the studies. In the McGovern et al. study, the pre- and post-localization task is a 2-IFC that resembles the 2-IFC simultaneity training task participants had to complete. In Odegaard et al.’s study, the pre- and post-test involved a simple audiovisual or unisensory localization task with a fairly different learning phase generally entailing passive observation of audiovisual stimuli while performing a fixation task and without explicit judgment about spatiotemporal relations. It is unlikely, however, that the attentional task demands from the active vs. passive multisensory learning trigger different learning mechanisms as this hypothesis cannot account for the results of Park and Kayser which did involve an active task [29]. Moreover, previous research suggests that selective attention simply does not have a significant effect on binding tendency in either temporal or spatial tasks [16].

The study by Tong and colleagues [30] also implicitly embraces the statistical learning framework. It is also difficult to reconcile their results with the results from Odegaard et al. [28] which found that predictive coding might more closely subserve binding tendency updates. One possible key difference between the two studies is that Tong and colleagues present two audiovisual stimulus pairs at once to participants in the exposure phase, whereas Odegaard et al. only present one pair at a time. Tong and colleagues posit that presenting two pairs at once may more closely reflect the natural environment where we may typically be simultaneously exposed to cross-modal inputs that are congruent and to cross-modal inputs that are incongruent [30]. More importantly, Tong and colleagues argue that this simultaneous presentation of a congruent stimulus pair and an incongruent stimulus pair, along with their use of a larger range of spatial disparities, is where the difference in results between their work and Odegaard et al.’s work lies. It is difficult to know with certainty which of the two frameworks the results of Tong and colleagues’ study conform to. Regardless, their findings suggest that binding tendency may depend on stimulus-feature level factors.

Future work should probe further the sources of variability in binding tendency. The work conducted hitherto varies greatly in its methods and involves several possible top-down and bottom-up factors that could intentionally or unintentionally influence the tendency to bind. Implications for statistical learning or predictive coding frameworks are difficult to make provided the paucity of work directly comparing these hypotheses. If evidence for both statistical learning-mediated binding tendency shifts and predictive coding-mediated shifts exist, how can we reconcile the divergent findings? It is important to recognize that the predictive coding and statistical learning accounts are not necessarily mutually exclusive and could operate under different conditions or even in parallel. One possibility is that statistical learning could operate in the early stages of audiovisual causal inference whereby an association is generated between not previously linked audiovisual signals. Once a link exists, predictive coding may be particularly important for adjusting the connection between audiovisual stimuli to account for prediction-error-driven adaptations to changing environment statistics. In other words, statistical learning could be especially relevant for establishing new audiovisual relationships whereas predictive coding may be relevant for adjusting causal expectations given new environmental conditions. The conditions under which binding tendency would be unchanged following passive or active learning also remain relatively unknown. Each of the discussed learning mechanisms leads to different explicit predictions about this question. As was outlined above, the statistical learning mechanism assumes that all sensory events are informative when it comes to binding tendency updates. Under this framework, every sensory event is considered evidence of a common cause or evidence of independent causes. Thereby, it could be that every sensory event influences binding tendency incrementally. Conversely, according to the predictive coding framework, only sensory events that violate the current expectations from the internal model of the world result in updates in binding tendency. Therefore, passive or active learning involving events that do not violate the internal model of the world should not result in binding tendency changes. Odegaard and colleagues offer some evidence for this by showing that exposure to spatio-temporally congruent audiovisual stimuli does not result in any changes to binding tendency.

## 6. Other Studies on Multisensory Perceptual Learning

In this section, we review other previous research that has explored the effects of multisensory training on multisensory processing and discuss their implications for plasticity in binding tendency.

Powers and colleagues tested whether simultaneity judgment training with trial-by-trial feedback could result in a narrowing of the temporal binding window [38]. Their results indicate a marked narrowing of the temporal binding window as well as stability in these training-induced effects seven days after the training session. They also observed a dilation of the temporal binding window for a control group that passively experienced temporally congruent stimuli in the majority of trials. They argue that because the proportion of simultaneous to non-simultaneous stimuli is larger during the exposure phase compared to the pre- and post-exposure assessments, the exposure phase increases the bias for congruency (or in other words, the binding tendency). This explanation is consistent with the statistical learning framework and could indeed underlie the observed widening of the temporal binding window. However, given that the same exact stimuli were used in the training condition, the same statistical regularity (proportion of simultaneous to non-simultaneous stimuli) was also present in that condition and should have given rise to a widening of the binding window, whereas the binding window narrowed in that condition. Therefore, we postulate the following account for their findings: Given the similarity of this study with that of McGovern and colleagues [27], it is likely that the simultaneity training caused an improvement of unisensory temporal precisions, as well as a reduction of binding tendency (as suggested by the analysis in McGovern et al. study) [27]. The reduction in binding tendency could be due to the top-down knowledge provided by feedback that the two signals are not related.

Arguably, because the aforementioned study employed BCI or measured unisensory precision before and after training, it is difficult to know for certain whether changes in unisensory reliability or binding tendency produced the observed results. In contrast to the above studies which involved temporal processing and explicit training of participants, a study by Wanrooij and colleagues investigated passive learning of binding tendency in a spatial localization task [39]. Reaction time (RT) to visual stimuli presented with congruent sounds (i.e., in congruent trials) was compared within participants between blocks with only congruent audiovisual stimuli vs. blocks with various proportions of congruent and incongruent audiovisual stimuli. RT was the lowest when congruent stimuli were presented in a block of all congruent trials compared to blocks wherein some trials were incongruent. Because the same type of unisensory stimuli was presented in all blocks, any improvement in unisensory precision can be assumed to be the same across blocks. The only difference between the blocks was the spatial relationship between the auditory and visual stimuli. Therefore, the difference in RT to the same stimulus within different contexts (different types of blocks) suggests that observers learned the statistical regularity of the AV relationships, through a statistical learning mechanism. However, as pointed out by Odegaard et al., the participants received discrepant instructions across blocks. For the fully congruent block, they received explicit knowledge about the fact that all the trials would be spatiotemporally aligned. This knowledge was not offered for the other blocks. Therefore, it is difficult to say with certainty that top-down factors did not intervene. Nonetheless, this study provides further evidence of rapid malleability of binding tendency.

A more recent study using a similar experimental design investigated the influence of prior sensory experiences on integration in a speech perception task [40]. Observers were presented with audiovisual movies of utterance of a syllable in each trial and were asked to report the syllable they perceived. In “congruent” blocks, the auditory and visual syllables were consistent in the majority of trials, whereas in “incongruent” blocks different syllables were presented in the audio and video simultaneously. In both blocks, the same exact incongruent (“McGurk stimuli”) trials were interspersed within the block [41]. Observers showed a stronger integration (i.e., experienced more McGurk illusion) in congruent blocks compared to incongruent blocks. These findings suggest that participants’ perceptual systems tracked and learned the relationship between the auditory and visual syllables and adjusted the binding tendency accordingly. This study appears to provide additional evidence for fast updates to binding tendency. However, it is worth noting that unisensory precision (assessed by either modeling, or by performance in unisensory trials) was not measured or estimated in the study, making it difficult to resoundingly conclude that the effect was entirely due to modulation of the binding tendency. These results also appear to be in conflict with those of Odegaard et al. [28]. In the work by Gau and Noppeney, repeated exposure to phonologically incongruent paired auditory-visual stimuli led to less integration, whereas in Odegaard et al.’s work, repeated exposure to spatially incongruent paired auditory-visual stimuli resulted in an increase in integration [28,40]. There are several differences in the experimental paradigms, however, that could have led to different learning mechanisms triggered in the two experiments. For example, in Gau and Noppeney’s study, participants were not passively exposed to the stimuli, and the degree and type of incongruence between the visual and auditory stimuli may have provided compelling evidence of independent causes, in contrast to the passive exposure and the limited spatial discrepancy in the Odegaard et al. study [28,40].

## 7. Neural Mechanisms

Bayesian causal inference has accounted for a wide range of perceptual and sensorimotor data (see [18] for a review). Recent neuroscientific studies have shed light on neural correlates of BCI and have suggested a distributed and hierarchical machinery in the human neocortex [42,43]. However, the neural circuitry underpinning causal inference computations in the nervous system is poorly understood. To disentangle these mechanisms in the context of multisensory integration plasticity, it is important to understand how the two central components of BCI, likelihood functions and prior probabilities of a common cause (i.e., *Pcommon*), are represented in the brain.

Evidence from previous studies has suggested that (1) manipulating likelihoods (via changes in sensory reliabilities) does not result in a change in priors and (2) conversely, manipulating priors (expectation of a common cause via repeated exposure to either congruent or incongruent audio-visual stimuli) does not result in a change in likelihoods [30,44]. These findings suggest that Bayesian likelihoods and priors are encoded independently from each other in the human nervous system. Here, we specifically focus on studies that explore the neural correlates of binding tendency (aka, *Pcommon* in the BCI framework; see section ‘Multisensory Binding Tendency’) and discuss mechanisms for encoding and updating the binding tendency.

Recent work has begun to explore the question of how prior information is stored at the neural level [45,46]. Generally, two types of prior information have been considered: the prior probabilities associated with unisensory estimates and the joint probability of two stimuli co-occurring. In terms of the unisensory prior information, some have proposed that this is represented by the density distribution of receptive fields where more frequent sensory events are represented by more dense neural populations. Regarding the binding tendency prior, it has been proposed that cross-modal synaptic connectivity could represent prior information where denser cross-modal synaptic connections could represent a greater tendency for audiovisual information to co-occur [46].

A recent model [46] utilizing these biologically inspired prior representations was successful at reproducing common multisensory integration illusions such as visual biasing of auditory localization estimates, known as the ventriloquist effect. The authors also explored the ability of the model in a mature state to re-learn input statistical regularities and re-adjust the prior representation through training. The Hebbian learning and a forgetting mechanism for perceptual regularities were sufficient to allow the model to update its density curves and cross-modal synaptic connectivity, which purportedly represents binding tendency.

While neural network models of multisensory perception have used excitatory connections between unisensory regions as a mechanism for implementing binding tendency and underlying the learning of joint cross-modal probabilities, crossmodal inhibitory connections can also possibly play a role in multisensory processing. A recent study in rodents suggests that long-range direct cortical circuits between the primary visual cortex (V1) and the auditory cortex (AuC) may serve as one of the main neural mechanisms for cross-modal predictive processing [47]. The study found that activity in a direct feedforward connection from AuC to V1 can be elicited by auditory signals and is independent of V1 activity [47]. More importantly, this connection functions by inhibiting V1 activity in response to *predictable* visual input. As the authors of the study discuss, the predictive processing model entails comparing bottom-up input to a top-down prediction. Prediction errors can involve signals that are unpredicted occurring or signals that *are* predicted *not* to occur. It is possible that these cross-modal long-range inhibitory connections are somehow involved in representing binding tendency at the neural level.

A recent study examined the neural correlates of binding tendency’s influence on multisensory integration in humans using fMRI. In this study [40], which was discussed earlier, brain activity was measured during an auditory-visual speech perception task in two different types of blocks that modulated the binding tendency. A significant interaction between the type of block the McGurk stimuli was interspersed in (congruent vs. incongruent) and whether or not the observers experienced the McGurk effect was found in the dorsal left inferior frontal sulcus (liFS). This finding was interpreted as dorsal liFS playing a role in combining binding tendency prior information with sensory information in the multisensory causal inference. However, the nature of the involvement of liFS in the representation of binding tendency remains unclear.

In the “Multisensory Associative Learning” section we discussed how forming associations between co-occurring sensory stimuli can lead to an increase in tendency to bind the stimuli. To investigate the neural changes induced by new associations, von Kriegstein and colleagues used fMRI to measure participants’ brain activity before, during, and after learning associations between voices and faces [31]. They found increased functional connectivity between voice and face areas as a result of such ecologically valid multisensory associative learning. A strengthened functional connectivity between two unisensory areas could represent increased prior probability.

**Figure 3:**
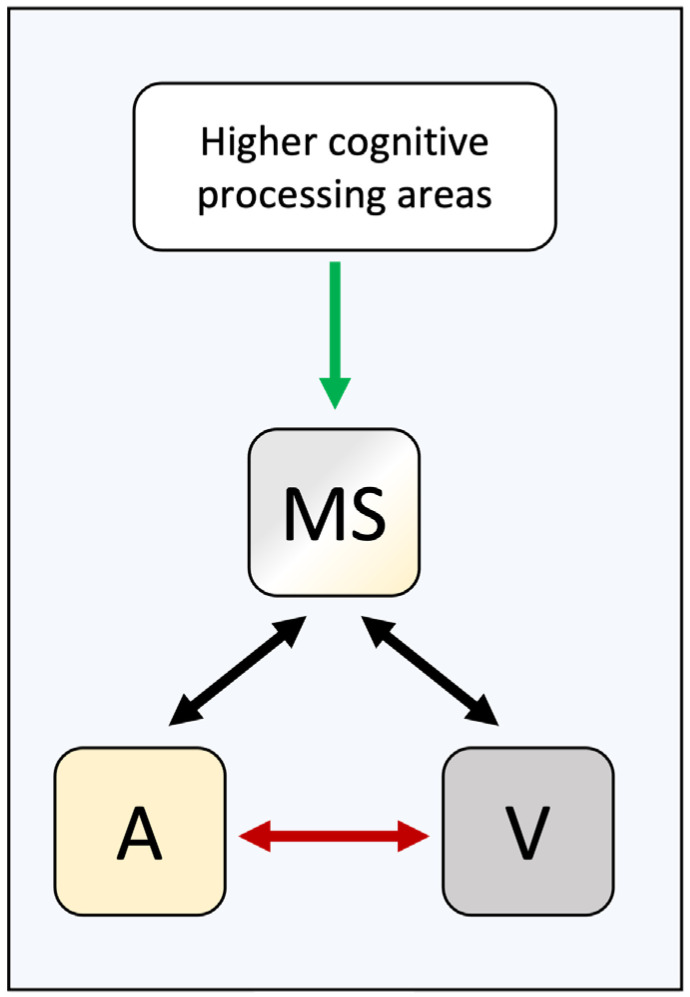
Possible neural substrates of binding tendency. The learned or hardwired association between the senses may be mediated by the lateral connectivity between unisensory regions, here denoted by the red arrow between auditory and visual regions. The strength of this connection would map to the degree of binding tendency. Extant models of multisensory integration have utilized this mechanism (see [45] as an example). Top-down influences on binding tendency may be mediated by projections from cognitive processing regions in the frontal lobe to multisensory processing areas involved in causal inference. This is denoted by the green arrow.

While these studies have started to shed light on some possible neural correlates of binding tendency, several basic questions still remain poorly understood or unsurveyed. For example, it is not clear whether top-down influences on binding tendency (such as cognitive knowledge) versus bottom-up influences (such as statistics of the stimuli in the environment) operate on different neural mechanisms and exert a bias on integration at different levels of processing. While the statistical models of integration such as BCI include a single variable (i.e., *Pcommon*) capturing the overall tendency to bind, it is conceivable that this abstract formulation has a more complex and distributed representation in the neural hierarchy of multisensory processing. If so, different learning mechanisms may also be involved in the plasticity of the different levels of binding prior. For example, it is conceivable that learned statistics of the stimuli (bottom-up information) leads to the adjustment of binding tendency via adjustment of lateral connectivity between unisensory areas, whereas cognitive (top-down) biases would influence binding tendency via modulation of activity in higher multisensory regions (Figure 3)**.**

## 8. Discussion

Any two emergent sensory signals either have a common cause or independent causes. If the nervous system infers a common cause, then the sensory cues should be integrated to result in the best estimate of their source. If the stimuli are determined to have independent causes, then they should be independently processed. This question of causal inference has been studied extensively in the context of unisensory properties of objects, also known as ‘within-modality binding’ [48]. In the context of unisensory processing, causal inference entails determining which set of properties of the same sensory modality belong to the same object. In multisensory processing, causal inference is the process of determining what information from distinct sensory modalities corresponds and originates from a common cause. According to the Bayesian framework, this process is shaped by the congruency between the sensory cues as well as the prior expectation of a common cause, aka, the tendency to bind.

The congruency between the sensory cues would be computed over all observable dimensions, including time, spatial location, as well as other dimensions such as semantics, and direction of motion if available. However, for any given set of sensory stimuli, the same participant may or may not infer a common cause depending on the prior expectation of a common cause (or “binding tendency”). In theory, this prior expectation or binding tendency can be formed partly by the statistics (i.e., the joint probability or co-occurrence) accrued from previous sensory events, which includes the perceptual history of the individual observer or the collective perceptual history accumulated over the course of evolution and hardwired in the perceptual system. In other words, based on our previous experiences, our brains probabilistically estimate how likely it is that, regardless of the current sensory information, specific cross-sensory inputs are associated and therefore ought to be integrated. Like many other sensory priors, in theory, the binding tendency can also be shaped by top-down factors such as cognitive knowledge, that could be explicitly provided (for example, by someone telling us that the stimuli are originating from the same object) or implicitly inferred (for example, by contextual cues).

In practice, the plasticity of binding tendency has been largely neglected in research. As a result, several basic questions regarding the binding tendency and its plasticity remain unanswered. Some of the most important ones are listed in Box 1 below. Some evidence suggests that binding tendency is relatively stable and domain- or task-specific [11,27,30]. It also appears that binding tendency is considerably malleable as studies discussed in the ‘Plasticity in Binding Tendency’ section report a change in binding tendency after a relatively limited amount of exposure or training. The few studies that have probed plasticity in binding tendency have varied greatly in paradigm, task, and stimuli. One reported a reduction in binding tendency following active multisensory learning with trial-by-trial feedback [27], while other studies showed that passive exposure to specific curated audiovisual events can be enough to increase binding tendency [28,38]. It seems that a single session lasting approximately a few minutes may be sufficient to induce a change in binding tendency [28]. Previous work also illustrates how subtle differences in instructions or experimental setup may also potentially influence binding tendency [19,27,39].

Box 1 **Outstanding questions**

1. How domain-specific, task-specific, and stimulus-specific is binding tendency?
2. How long do changes in binding tendency last?
3. What are the neural correlates of binding tendency?
4. How fast does binding tendency change? How much training or exposure is required to obtain a measurable shift?
5. What are the underlying learning mechanisms involved in binding tendency plasticity?
6. Can predictive coding, statistical learning, or both account for changes in binding tendency? If so, under what conditions would each operate?
7. What are the exposure or training protocols that would increase the binding tendency? What are the exposure or training protocols that would decrease the binding tendency?
8. Are temporal relationships more critical than spatial relationships in manipulating binding tendency?
9. What is the size of the window of perceptual history referenced for estimating the prior probability of common cause? Are more recent events given a bigger weight in this window?

Importantly, the ‘recipe’ for *how* to systematically shift binding tendency is currently unknown. There are two interrelated specific open questions here. First, what are the learning mechanisms that mediate updating of binding tendencies? Second, what are the specific stimulus conditions and their necessary presentation frequency that induce the binding tendency to increase or decrease? Two emergent frameworks from neuroscience at large and from the research discussed here offer some candidate propositions for the underlying mechanisms. One is statistical learning of joint probabilities of the sensory stimuli. Another one is correction of the prediction errors because of inconsistencies between sensory experience and the internal model prediction. These frameworks can produce distinct strategies for modifying binding tendency. As discussed in the section ‘Learning Mechanisms involved in Binding Plasticity’, previous studies have provided support for both mechanisms. These mechanisms are not necessarily mutually exclusive and may both operate in parallel. Future research will need to shed light on conditions that promote one versus the other, and whether they can both be harnessed simultaneously and synergistically. More research should be conducted, especially focusing on the role of surprise-minimization and other *types* of surprise in the context of learning-elicited multisensory prediction error [49]. Future research should also investigate whether the neural mechanisms that appear to underlie cross-modal predictive processing may also be related to audiovisual binding tendency representation in the brain [47].

Although previous research discussed here has shed light on the nature of binding tendency malleability, many open questions remain with regards to several aspects of this important ingredient of multisensory integration and causal inference (see Box 1). For instance, although audiovisual binding tendency seems to be reshaped with passive audiovisual exposure in the span of less than an hour, it remains unclear how permanent these changes are. No studies have systematically examined how long shifts in binding tendency last. One possibility could be that individuals all have a baseline binding tendency established by our regular perceptual environment and any binding tendency shifts eventually revert back to our baseline. Another possibility could be that binding tendency shifts are task and context specific and are long lasting in their context. Future studies should probe the question of permanence further. Moreover, the window of perceptual history whose statistics shape the prior probability of a common cause remains unknown. Even if the size of the window is infinite and all the perceptual history is taken into account, it is possible that more recent events have a stronger influence on the binding tendency than very old events. The size of this perceptual history may also change over time, it may be that given certain contextual or stimulus-type factors, the nervous system references more of our perceptual history in some cases and less in others.

More work focusing on how *much* training is necessary to cause a shift, in particular, the minimal amount necessary and when these multisensory learning effects may or may not plateau needs to be conducted. Audiovisual statistical learning can occur fairly rapidly, which suggests that the amount of learning necessary to shift binding tendency may be fairly small in general. However, more work on the impacts of the speed of learning on the magnitude and durability of change this elicits in binding tendency needs to be considered.

One important finding that needs to be examined further is the fact that temporal congruence may be a stronger cue for signaling a common cause than spatial congruence [28]. This finding makes sense, if the perceptual system keeps track of joint probabilities which by definition require the co-occurrence of events in time. Moreover, it has recently been proposed that the nervous system may use the temporal correlation between the sensory signals to carry out multisensory causal inference [50,51]. This is consistent with the findings of Odegaard et al. [28] that suggested a critical role of repeated temporal co-occurrence of auditory-visual stimuli in providing compelling evidence of common cause (despite the large spatial incongruency) and leading to an increase in binding tendency. Having said that, little research has rigorously questioned the unique affordances of spatial and temporal integration cues in shifting binding tendency and to what extent these affordances depend on the task at hand or the current perceptual milieu. Most of the recent multisensory learning studies have mainly used spatial judgment tasks to assess binding tendency. No work has investigated the effects of multisensory learning on binding tendency in a temporal numerosity task, for example. One previous study looked at the stability of binding tendency in a temporal numerosity judgment task; however, the plasticity of binding tendency was outside of the scope of the study [11].

Importantly, as was mentioned earlier, a study testing the same participants in both a spatial and temporal task found that the respective binding tendencies during a temporal numerosity task and spatial localization task were divergent [11]. Previous research discussed in this review also unveils another important open question about binding tendency [28,30]. If binding tendency is different for temporal and spatial tasks and even within the same task for different audiovisual stimulus pairs, what does this mean for how the binding tendency is encoded in the perceptual system and parameterized in the BCI model?

Finally, the neural correlates of binding tendency remain elusive. Neural network modeling has suggested the strength of lateral connectivity between unisensory regions as one possible neural mechanism and human neuroimaging studies have reported an increase in functional connectivity between unisensory brain regions following associative crossmodal learning [31,46]. Further research is needed to unravel the neural correlates of binding tendency in different processing domains, and for different types of sources of bias, namely top-down vs. bottom-up binding priors.

Over the last two decades, multisensory research has exploded, and one of the more recent areas of inquiry in this domain has been the question of how multisensory processing adapts to the statistical properties of the environment. Extensive research has uncovered various ways in which audiovisual processing unfolds. However, the mechanisms underlying causal inference in multisensory integration have been ultimately derelict. Some recent progress has been made to uncover the mysteries of the tendency to bind the senses. However, much more work has to be done before we can fully understand this paramount stage of the multisensory perceptual process. Some researchers have suggested there is a deficiency in multisensory integration in dyslexia [52], autism spectrum disorder [53,54], and schizophrenia [55,56]. Understanding mechanisms and gaining insight into protocols for enhancing multisensory integration can potentially contribute to the development of remedial strategies for these disorders.

## Data Availability

Not applicable.
