# Correction: Quintero et al. Changing the Tendency to Integrate the Senses. *Brain Sci.* 2022, *12*, 1384

**DOI:** 10.3390/brainsci14101011

**Published:** 2024-10-09

**Authors:** Saul I. Quintero, Ladan Shams, Kimia Kamal

**Affiliations:** 1 Department of Psychology, University of California, Los Angeles, CA 90095, USA; 2 Department of Bioengineering, University of California, Los Angeles, CA 90089, USA; 3 Neuroscience Interdepartmental Program, University of California, Los Angeles, CA 90089, USA

In the original publication [1], there was a mistake in Figure 2 as published. Figure 2 describes the learning mechanisms involved in binding tendency plasticity. Figure 2 as published includes two mechanisms: (a) the statistical learning mechanism, and (b) the predictive coding framework. According to the predictive coding framework, illustrated in Figure 2b, only sensory events that contradict the internal generative model of the sensory environment will elicit updates in the prior probability of a common cause. 

For instance, if the internal generative model of the world expects that incongruent stimuli do not have a common cause, and an observer repeatedly experiences spatially incongruent but temporally congruent stimuli, these repeated sensory experiences contradict the internal model of the world and produce a model prediction-error signal. This results in a relaxation of binding tendency and an increased probability of common cause to account for the congruent stimuli that are nonetheless spatially discrepant. Figure 2 as published mistakenly lists the binding tendency change, resulting from spatially incongruent stimuli presented as temporally congruent, as a reduction in binding tendency. This is incorrect as the result should be an increase in the binding tendency as correctly described in the figure description. The corrected Figure 2 appears below. 

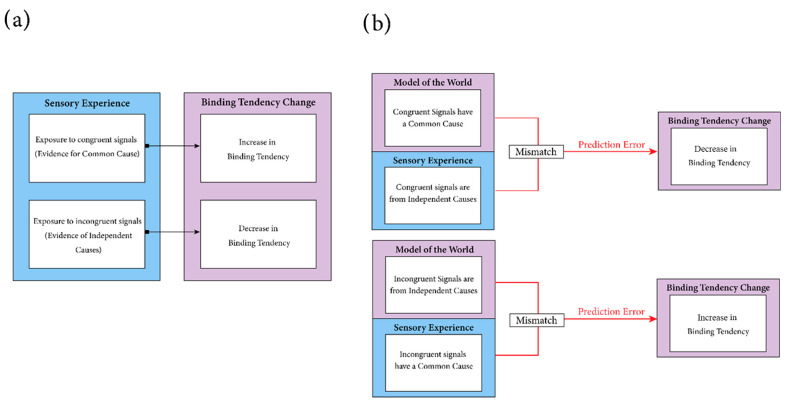



The authors state that the scientific conclusions are unaffected. This correction was approved by the Academic Editor. The original publication has also been updated.

## References

[1] Quintero S.I., Shams L., Kamal K. (2022). Changing the Tendency to Integrate the Senses. Brain Sci..

